# Ten Simple Rules for Digital Data Storage

**DOI:** 10.1371/journal.pcbi.1005097

**Published:** 2016-10-20

**Authors:** Edmund M. Hart, Pauline Barmby, David LeBauer, François Michonneau, Sarah Mount, Patrick Mulrooney, Timothée Poisot, Kara H. Woo, Naupaka B. Zimmerman, Jeffrey W. Hollister

**Affiliations:** 1 University of Vermont, Department of Biology, Burlington, Vermont, United States of America; 2 University of Western Ontario, Department of Physics and Astronomy, London, Canada; 3 University of Illinois at Urbana-Champaign, National Center for Supercomputing Applications and Institute for Genomic Biology, Urbana, Illinois, United States of America; 4 University of Florida, iDigBio, Florida Museum of Natural History, Gainesville, Florida, United States of America; 5 University of Florida, Whitney Laboratory for Marine Bioscience, Gainesville, Florida, United States of America; 6 King’s College London, Department of Informatics, London, United Kingdom; 7 University of California at San Diego, San Diego Supercomputer Center, San Diego, California, United States of America; 8 Université de Montréal, Département de Sciences Biologiques, Montreal, Canada; 9 Washington State University, Center for Environmental Research, Education, and Outreach, Pullman, Washington, United States of America; 10 University of Arizona, School of Plant Sciences, Tucson, Arizona, United States of America; 11 US Environmental Protection Agency, Atlantic Ecology Division, Narragansett, Rhode Island, United States of America; Dassault Systemes BIOVIA, UNITED STATES

## Introduction

Data is the central currency of science, but the nature of scientific data has changed dramatically with the rapid pace of technology. This change has led to the development of a wide variety of data formats, dataset sizes, data complexity, data use cases, and data sharing practices. Improvements in high-throughput DNA sequencing, sustained institutional support for large sensor networks [1,2], and sky surveys with large-format digital cameras [3] have created massive quantities of data. At the same time, the combination of increasingly diverse research teams [4] and data aggregation in portals (e.g., for biodiversity data, GBIF.org or iDigBio) necessitates increased coordination among data collectors and institutions [5,6]. As a consequence, “data” can now mean anything from petabytes of information stored in professionally maintained databases, to spreadsheets on a single computer, to handwritten tables in lab notebooks on shelves. All remain important, but data curation practices must continue to keep pace with the changes brought about by new forms of data and new data collection and storage practices.

While much has been written about both the virtues of data sharing [7,8] and the best practices to do so [9,10], data storage has received comparatively less attention. Proper storage is a prerequisite to sharing, and indeed inadequate storage contributes to the phenomenon of data decay or to “data entropy,” in which data, whether publicly shared or not, becomes less accessible through time [11–14]. Best practices for data storage often begin and end with this statement: “Deposit your data in a community standard repository.” This is good advice, especially considering your data is most likely to be reused if it is available on a community site. Community repositories can also provide guidance for best practices. As an example, if you are archiving sequencing data, a repository such as those run by the National Center for Biotechnology Information (NCBI) (e.g., GenBank) not only provides a location for data archival but also encourages a set of practices related to consistent data formatting and the inclusion of appropriate metadata. However, data storage policies are highly variable between repositories [15]. A data management plan utilizing best practices across all stages of the data life cycle will facilitate transition from local storage to repository [16]. Similarly, having such a plan can facilitate transition from repository to repository if funding runs out or requirements change. Good storage practices are important even (or especially) in cases when data may not fit with an existing repository, when only derived data products (versus raw data) are suitable for archiving, or when an existing repository may have lax standards.

This article describes ten simple rules for digital data storage that grew out of a long discussion among instructors for the Software and Data Carpentry initiatives [17,18]. Software and Data Carpentry instructors are scientists from diverse backgrounds who have encountered a variety of data storage challenges and are active in teaching other scientists best practices for scientific computing and data management. Thus, this paper represents a distillation of collective experience, and hopefully will be useful to scientists facing a variety of data storage challenges. We additionally provide a glossary of common vocabulary for readers who may not be familiar with particular terms.

## Rule 1: Anticipate How Your Data Will Be Used

One can avoid most of the troubles encountered during the analysis, management, and release of data by having a clear roadmap of what to expect before data acquisition starts. For instance:

- How will the raw data be received? Are they delivered by a machine or software, or typed in?
- What is the format expected by the software used for analysis?
- Is there a community standard format for this type of data?
- How much data will be collected, and over what period of time?

The answers to these questions can range from simple cases (e.g., sequencing data stored in the FASTA format, which can be used “as is” throughout the analysis), to experimental designs involving multiple instruments, each with its own output format and processing conventions. Knowing the state in which the data needs to be at each step of the analysis can help to (i) identify software tools to use in converting between data formats, (ii) orient technological choices about how and where the data should be stored, and (iii) rationalize the analysis pipeline, making it more amenable to re-use [19].

Also key is the ability to estimate the storage volume needed to store the data, both during and after the analysis. The required strategy will differ for datasets of varying size. Smaller datasets (e.g., a few megabytes in size) can be managed locally with a simple data management plan, whereas larger datasets (e.g., gigabytes to petabytes) will in almost all cases require careful planning and preparation (Rule 10).

Lastly, early consideration and planning should be given to the metadata of the project. A plan should be developed early as to what metadata will be collected and how it will be maintained and stored (Rule 7). Also be sure to consider community software tools that can facilitate metadata curation and repository submission. Examples in the biological sciences include *Morpho* for ecological metadata [20] and *mothur* [21] for submitting to NCBI’s Sequence Read Archive.

## Rule 2: Know Your Use Case

Well-identified use cases make data storage easier. Ideally, prior to beginning data collection, researchers should be able to answer the following questions:

- Should the raw data be archived (Rule 3)?
- Should the data used for analysis be prepared once or re-generated from the raw data each time (and what difference would this choice make for storage, computing requirements, and reproducibility)?
- Can manual corrections be avoided in favor of programmatic or self-documenting approaches (e.g., Jupyter notebook or R markdown)?
- How will changes to the data be tracked, and where will these tracked changes be logged?
- Will the final data be released, and if so, in what format?
- Are there restrictions or privacy concerns associated with the data (e.g., survey results with personally identifiable information [PII], threatened species, or confidential business information)?
- Will institutional validation be required prior to releasing the data?
- Does the funding agency mandate data deposition in a publicly available archive, and if so, when, where, and under what license?
- Does the target journal mandate data deposition?

None of these questions have universal answers, nor are they the only questions to ask before starting data acquisition. But knowing the what, when, and how of your use of the data will bring you close to a reliable roadmap on how to handle data from acquisition through publication and archival.

## Rule 3: Keep Raw Data Raw

Since analytical and data processing procedures improve or otherwise change over time, having access to the “raw” (unprocessed) data can facilitate future re-analysis and analytical reproducibility. As processing algorithms improve and computational power increases, new analyses will be enabled that were not possible at the time of the original work. If only derived data are stored, it can be difficult for other researchers to confirm analytical results, to assess the validity of statistical models, or to directly compare findings across studies.

Therefore, data should always be kept in raw format whenever possible (within the constraints of technical limitations). In addition to being the most appropriate way to ensure transparency in analysis, having the data stored and archived in their original state gives a common point of reference for derivative analyses. What constitutes sufficiently “raw” data is not always clear (e.g., ohms from a temperature sensor or images of an Illumina sequencing flowcell are generally not archived after the initial processing). Yet the spirit of this rule is that data should be as “pure” as possible when they are stored. If derivations occur, they should be documented by also archiving relevant code and intermediate datasets.

A cryptographic hash (e.g., SHA or MD5) of the raw data should be generated and distributed with the data. These hashes ensure that the dataset has not suffered any silent corruption and/or manipulation while being stored or transferred (see Internet2 Silent Data Corruption). For large enough datasets, the likelihood of silent data corruption is high. This technique has been widely used by many Linux distributions to distribute images and has been very effective with minimal effort.

## Rule 4: Store Data in Open Formats

To maximize accessibility and long-term value, it is preferable to store data in formats that have freely available specifications. The appropriate file type will depend on the data being stored (e.g., numeric measurements, text, images, video), but the key idea is that accessing data should not require proprietary software, hardware, or purchase of a commercial license. Proprietary formats change, maintaining organizations go out of business, and changes in license fees make access to data in proprietary formats unaffordable and risky for end-users. Examples of open data formats include comma-separated values (CSV) for tabular data, hierarchical data format (HDF) [22] and NetCDF [23] for hierarchically structured scientific data, portable network graphics (PNG) for images, KML (or other Open Geospatial Consortium [OGC] format) for spatial data, and extensible markup language (XML) for documents. Examples of closed formats include DWG for AutoCAD drawings, Photoshop document (PSD) for bitmap images, Windows Media Audio (WMA) for audio recording files, and Microsoft Excel (XLS) for tabular data. Even if day-to-day processing uses closed formats (e.g., due to software requirements), data being stored for archival purposes should be stored in open formats. This is generally not prohibitive; most closed-source software products enable users to export data to an open format.

Not only should data be stored in an open format but it should also be stored in a format that computers can easily use for processing. This is especially crucial as datasets become larger. Making data easily usable is best achieved by using standard data formats that have open specifications (e.g., CSV, XML, JSON, HDF5), or by using databases. Such data formats can be handled by a variety of programming languages, as efficient and well-tested libraries for parsing them are typically available. These standard data formats also ensure interoperability, facilitate re-use, and reduce the chances of data loss or mistakes being introduced during conversion between formats. Examples of machine-readable open formats that would not be easy to process include data included in the text of a PDF file or scanned images of tabular data from a paper source.

## Rule 5: Data Should Be Structured for Analysis

**Fig 1:**
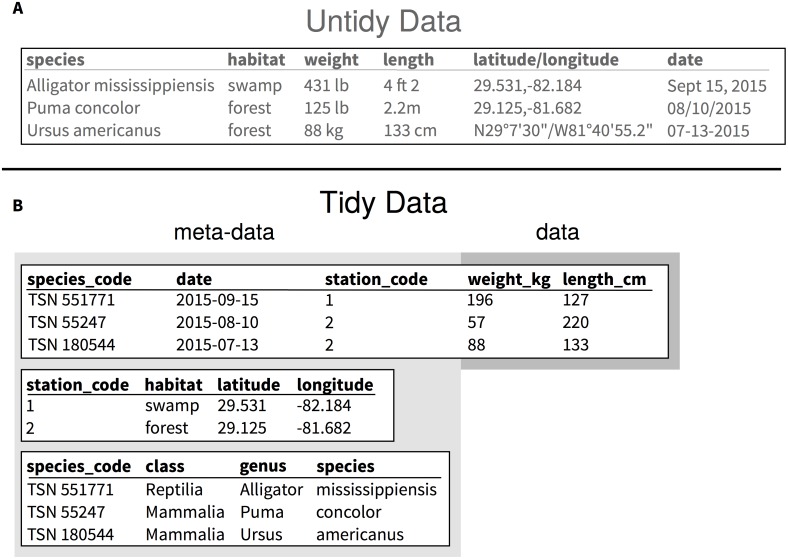
Example of an untidy dataset (A) and its tidy equivalent (B). Dataset A is untidy because it mixes observational units (species, location of observations, measurements about individuals), the units are mixed and listed with the observations, more than one variable is listed (both latitude and longitude for the coordinates, and genus and species for the species names), and several formats are used in the same column for dates and geographic coordinates. Dataset B is an example of a tidy version of dataset A that reduces the amount of information that is duplicated in each row, limiting chances of introducing mistakes in the data. By having species in a separate table, they can be identified uniquely using the Taxonomic Serial Number (TSN) from the Integrated Taxonomic Information System (ITIS), and it makes it easy to add information about the classification of these species. It also allows researchers to edit the taxonomic information independently from the table that holds the measurements about the individuals. Unique values for each observational unit facilitate the programmatic combination of information using “join” operations. With this example, if the focus of the study for which these data were collected is based upon the size measurements of the individuals (weight and length), information about “where,” “when,” and “what” animals were measured can be considered metadata. Using the tidy format makes this distinction clearer.

To take full advantage of data, it can be useful for it to be structured in a way that makes use, interpretation, and analysis easy. One such structure for data stores each variable as a column, each observation as a row, and each type of observational unit as a table (Fig 1). The technical term for this structure is “Codd’s 3rd normal form,” but it has been made more accessible as the concept of tidy data [24]. When data is organized in this way, the duplication of information is reduced and it is easier to subset or summarize the dataset to include the variables or observations of interest.

One axiom about the structure of data and code holds that one should “write code for humans, write data for computers” [25]. When data can be easily imported and manipulated using familiar software (whether via a scripting language, a spreadsheet, or any other computer program that can import these common files), data becomes easier to re-use. Furthermore, having the source code for the software doing the analysis available provides provenance for how the data is processed and analyzed. This makes analysis more transparent, since all assumptions about the structure of the data are implicitly stated in the source code. This also enables extraction of the analyses performed, their reproduction, and their modification.

Interoperability is facilitated when variable names are mapped to existing data standards. For instance, for biodiversity data, the Darwin Core Standard provides a set of terms that describe observations, specimens, samples, and related information for a taxa. For earth science and ecosystem models and data, the Climate Forecasting Conventions are widely adopted, such that a large ecosystem of software and data products exist to reduce the technical burden of reformatting and reusing large and complex data. Because each term in such standards is clearly defined and documented, each dataset can use the terms consistently; this facilitates data sharing across institutions, applications, and disciplines. With machine-readable, standards-compliant data, it becomes easier to build an Application Programming Interface (API) to query the dataset and retrieve a subset of interest, as outlined in Rule 10.

## Rule 6: Data Should Be Uniquely Identifiable

To aid reproducibility, the data used in a scientific publication should be uniquely identifiable. Ideally, datasets should have a unique identifier such as a Digital Object Identifier (DOI), Archival Resource Key (ARK), or a persistent URL (PURL). An increasing number of online services, such as Figshare, Zenodo, or DataOne, are able to provide these. Institutional initiatives also exist and are known to your local librarians. Some repositories may require specific identifiers, and these could change with time. For instance, NCBI sequence data will in the future only be identified by “accession.version” IDs. The “GI” identifiers (in use since 1994) will be retired in late 2016 [26].

Even as identifier standards may change over time, datasets can evolve over time as well. In order to distinguish between different versions of the same data, each dataset should have a distinct name, which includes a version identifier. A simple way to do this is to use date stamps as part of the dataset name. Using the ISO 8601 standard avoids regional ambiguities: it mandates the date format YYYY-MM-DD (i.e. from largest time unit to smallest). For example, the date “February 1, 2015,” while written as 01-02-2015 in the UK and 02-01-2015 in the US, is unambiguous (2015-02-01) under this standard.

Semantic versioning is a richer approach to solving the same problem [27]. The CellPack datasets are an example of this [28]. A semantic version number takes the form: Major.Minor.Patch, e.g., 0.2.7. The *major version* numbers should be incremented (or bumped) when a dataset scheme has been updated or some other change is made that is not compatible with previous versions of the data with the same major version number. This means that an experiment using version 1.0.0 of the dataset may not run on version 2.0.0 without changes to the data analysis. The *minor version* should be bumped when a change has been made that is compatible with older versions of the data with the same major version. This means that any analysis that can be performed on version 1.0.0 of the data is repeatable with version 1.1.0 of the data. For example, adding a new year in a temporal survey will result in a bump in the minor version. The *patch version* number is bumped when typos or bugs have been fixed. For example version 1.0.1 of a dataset may fix a typo in version 1.0.0.

## Rule 7: Link Relevant Metadata

Metadata is the contextual information required to interpret data (Fig 1) and should be clearly defined and tightly integrated with data. The importance of metadata for context, reusability, and discovery has been written about at length in guides for data management best practices [9,13,29].

Metadata should be as comprehensive as possible, using standards and conventions of a discipline, and should be machine-readable. Metadata should always accompany a dataset, wherever it is stored, but the best way to do this depends on the format of the data. Text files can contain metadata in well-defined text files such as XML or JSON. Some file formats are self-documenting; for example, NetCDF, HDF5, and many image file formats allow for embedded metadata [22,23]. In a relational database, metadata tables can be clearly labeled and linked to the data. Ideally, a schema will be provided that also shows the linkages between data tables and metadata tables. Another—simpler—scenario is a set of flat (non-hierarchical) text files—in this case a semantically versioned, compressed archive should be created that includes metadata.

Whatever format is used for archiving, the goal should be to make the link between metadata and data as clear as possible. The best approach is dependent on the archiving plan used, but even if the dataset is archived solely for personal use, metadata will provide crucial context for future reuse.

## Rule 8: Adopt the Proper Privacy Protocols

In datasets for which privacy is important, be sure to have a plan in place to protect data confidentiality. You should consider the different data stakeholders when developing privacy protocols for your data storage. These stakeholders include funding agencies, human subjects or entities, collaborators, and yourself. Both the United States National Science Foundation and National Institutes of Health have data sharing policies in their grant guidelines to prevent sharing personally identifiable information and to anonymize data on human subjects.

In small datasets, a lookup table (protecting PII by removing it and replacing it with a unique ID that maps to the sensitive data in an external dataset) is enough to anonymize a minimal amount of personal information. Hashing techniques are susceptible to a number of attacks, and all hashed data can eventually be determined. Famously, New York City officials shared what they thought was anonymized data on cab drivers and over 173 million cab rides. However, it was quickly recognized that the city anonymized the data with a simple MD5 hashing scheme and all 20 GB of data were de-anonymized in a matter of hours [30]. This type of error can be prevented by asking a trusted colleague or security personnel to try to “crack” anonymised data before releasing it publicly. Often the person who has produced the data is not well placed to check the fine details of their own security procedures. If possible, the best solution is to remove any sensitive data that is not required from the dataset prior to distribution.

In more problematic cases, the data itself allows identifiability: this is the case with human genomic data that map directly onto a subject’s identity [31]. Methods for dealing with these complex issues at the intersection of data storage and privacy are rapidly evolving and include storing changes against a reference genome to help with privacy and reduce overall data volumes [32,33] and/or bringing computation to data storage facilities instead of vice versa [34]. Having a plan for privacy before data is acquired is important because it can determine or limit how data will be stored.

## Rule 9: Have a Systematic Backup Scheme

Every storage medium can fail, and every failure can result in loss of data. Researchers should therefore back up data at all stages of the research process. Data stored on local computers or institutional servers during the collection and analysis phases of a project should be backed up to other locations to protect against data loss. No backup system is failsafe (see the stories of the Dedoose crash and the near deletion of Toy Story 2), so more than one backup system should be used. Ideally you should have two on-site copies (such as on a computer, an external hard drive, or a tape) and one off-site copy (e.g., cloud storage) [35], with care taken to ensure that the off-site copy is as secure as the on-site copies. Keeping backups in multiple locations additionally protects against data loss due to theft or natural disasters.

Researchers should test their backups regularly to ensure that they are functioning properly. Common reasons for backup failure include:

- faulty backup software
- incorrect configuration (e.g., not backing up sub-directories)
- encryption (e.g., someone encrypted the backups but later lost the password to decrypt them)
- media errors

Consider the backup plan of your selected data repository before publishing your data and if possible, find out about the long-term storage plans of the repository. Many repositories mirror the data they host on multiple machines. Are there plans in place to keep data available if the organization that manages the repository dissolves?

## Rule 10: The Location and Method of Data Storage Depend on How Much Data You Have

The storage method you should choose depends on the size and nature of your data, the cost of storage and access over time, the time it takes to transfer the data, how the data will be used, and any privacy concerns. Data is increasingly generated in the range of many terabytes (TB) by environmental sensors, satellites, automated analytical tools, simulation models, and nucleic acid sequencers. Even larger data-generating machines, like the Large Hadron Collider (LHC) and the Large Scale Synoptic Survey Telescope (LSST), generate many TB per day, rapidly accumulating to petabyte (PB) scale over the course of any particular study. While the cost of storage continues to decrease, the volume of data to be stored impacts the choice of storage methods and locations: for large datasets it is necessary to balance the cost of storage with the time of access and costs of re-generating the data. With new commercial cloud offerings (e.g., Amazon S3) the cost of retrieving the data might exceed the cost of analysis or of re-generating the data from scratch.

When data takes too long to transfer or is costly to store, it can become more efficient to use a computer system for analysis that can directly access and use the data in place instead of first transferring it to a local machine. Inactive data can be put in longer-term storage; this is less expensive, but can take longer to retrieve. Some storage systems automatically migrate “stale” files to longer-term storage. Alternatively, some computing can be done “in the database” or “on disk” via database query languages (e.g., SQL, MapReduce) that perform basic arithmetic, or via the use of procedural languages (e.g., R, Python, C) embedded in the database server. Modern database technologies such as HDFS and Spark allow these computations to be done on data of almost any size. When data is larger than locally available RAM, it can be handled by conducting analyses on a “big memory” node, which most high-performance computing centers have deployed. Relying on tight software/hardware integration, these can allow for the analysis of datasets around 1–4 TB in size. This allows the user to read in and use a large dataset without special tools.

If you regularly only need access to a small subset of your data or need to share it with many collaborators, a web-based API (Application Programming Interface) might be a good solution. Using this method, many users can send requests to an online service that can subset the data, perform in-database computation, and return smaller volumes of data as specific slices. Tools based on online services make it easier to find and download data, and they facilitate analysis via reproducible scripts. However, they can also lead to excessive and careless abuse of resources without proper safeguards in place. The time required to re-download and re-compute results can be reduced by “caching.” Caching stores copies of downloads and generated files that are recognized when the same script is run multiple times.

## Further Reading and Resources

Digital data storage is a vast topic; the references given here and elsewhere in this paper provide some starting points for interested readers. For beginning users of scientific data, Data Carpentry offers workshops and resources on data management and analysis, as do the DataONE education modules [36]. For librarians and others who are responsible for data archiving, Data Curation Profiles [37] may be of interest.

## Glossary

### Projects and initiatives

- **Global Biodiversity Information Facility** (GBIF, http://www.gbif.org) provides an international open data infrastructure to publish and disseminate biodiversity information.
- **Integrated Digitized Biocollections** (iDigBio, https://www.idigbio.org) is a project funded by the National Science Foundation that facilitates the digitization of natural history collections and provides data and images for biological specimens.
- **Integrated Taxonomic Information System** (ITIS, http://www.itis.gov) is an international partnership of governmental organizations that aims at providing authoritative taxonomic information for plants, animals, fungi, and microbes.

### File formats

- **Comma-Separated Values** (CSV) and **Tab-Separated Values** (TSV) are plain text file formats used to store tabular data, in which each row is represented by a line in the file and each field (column) is separated by a comma (for CSV) or by the tab character (for TSV).
- **FASTA** is a simple and widely used file format used to represent sequences of nucleotides or amino acids in plain text, making it easy to manipulate these programmatically.
- **Hierarchical Data Format** (HDF) is an open-source binary file format designed to store large amounts of data (and their associated metadata) by providing a hierarchical structure that could be compared to how a hard drive is organized with directories and files. It is maintained by the non-profit HDF Group, a spin-off of the National Center for Supercomputing Applications (NCSA).
- **JavaScript Object Notation** (JSON) is a plain text file format typically used to store arbitrarily structured data in the form of keys and values. It can be used to store non-relational databases, as it does not rely on a tabular data format. In many respects, it has been replacing XML.
- **Network Common Data Form** (NetCDF) is an open-source binary file format designed to store large datasets in array-oriented scientific data, typically used in the geosciences. It is maintained by Unidata, a non-profit member of the University Corporation for Atmospheric Research (UCAR), which is funded by the National Science Foundation.
- **Extensible Markup Language** (XML) is a markup language and the file format used to store documents written with it. It is used to represent arbitrary data structures and is both human and machine-readable.

### Programming and algorithms

- **Web Application Programming Interface** (API) provide ways to programmatically query databases through the internet. They notably allow users to retrieve and work with a small slice of a large dataset.
- **Hadoop Distributed File System** (HDFS) is a Java-based file system in which data is stored in small chunks across multiple redundant nodes.
- **MapReduce** is a style of programming designed to work with large datasets in parallel computing environments. Such programs are composed of a **map** procedure in which the dataset is sliced into several pieces, and a **reduce** procedure in which summary operations are then applied to each of the slices.
- **Secure Hash Algorithm 2** (SHA-2) is a family of Secure Hashing Algorithms used in cryptographic analysis, often to verify the integrity of a file. A cryptographic hash function converts a “message” (e.g., passwords, file content) into an encrypted value. Cryptographic hash functions are easy to compute from the message, but it should be impossible to recover the message from the output, and any modifications to the message should also modify the output. The SHA algorithms are often used in preference to similar tools such as MD5 (mentioned in Rule 3 and in Rule 8), which are no longer secure. All hashing algorithms are vulnerable to brute force attacks. Key Derivation Function (KDF) implementations like BCrypt and PBKDF2 are considered significantly more secure, but by design more costly to compute.
- Apache **Spark** is an open-source computing platform for querying large datasets in memory, in contrast to on-disk–based methods like MapReduce.
- **Structured Query Language** (SQL) is a programming language used to interact with relational database management systems.

### Hardware

- **mega-, giga-, tera-, petabytes** are units of digital information and are used to measure the size of datasets or the storage media. Originally a byte was the minimum amount of memory needed to store a single character of text in a computer. The prefixes mega-, giga-, tera-, and peta- refer to the international system of units for the multiple of the unit and correspond to 10^6^, 10^9^, 10^12^, and 10^15^, abbreviated M, G, T, and P, respectively.

### Persistent identifiers

- **Archival Resource Key** (ARK) identifiers are URLs designed to support long-term access to information online.
- **Digital Object Identifier** (DOI) provides unique and persistent identifiers for electronic documents (in particular, journal articles and datasets) on the internet. The uniqueness of the identifiers is guaranteed by a central registry. By dissociating the identifier and the location of the document (i.e., the URL), the DOI can remain fixed even if the location of the digital object it is pointing to changes.
- **Persistent Uniform Resource Locator** (PURL) is a URL used to redirect to the location of an electronic object on the internet. DOI and ARK are examples of implementations of PURL.
- **Uniform Resource Locator** (URL) gives the location of an object on the World Wide Web; the most familiar type of URL is a website address.
